# 185. Does an Infectious Diseases Consultation Improve Clinical Outcomes and Treatment Bundle Adherence for Enterococcal Bacteremia in a Multicenter Healthcare System?

**DOI:** 10.1093/ofid/ofab466.387

**Published:** 2021-12-04

**Authors:** Emily A Shephard, Kristin E Mondy, Kelly R Reveles, Theresa Jaso, Dusten T Rose

**Affiliations:** 1 Dell Seton Medical Center, Austin, Texas; 2 University of Texas Dell Medical School, Austin, Texas; 3 University of Texas at Austin, San Antonio, TX; 4 Ascension Texas, Austin, TX; 5 Seton Healthcare Family, Austin, TX

## Abstract

**Background:**

Infectious diseases consultation (IDC) for *Staphylococcus aureus* bacteremia has a known mortality benefit, but for other gram positive bacteremias the benefit is not known. This study examined differences in outcomes for enterococcal bacteremia when management includes IDC.

**Methods:**

This retrospective multicenter observational cohort study included adults with at least 1 positive blood culture with *Enterococcus* species. Patients who died or transferred to palliative care within 2 days of positive blood cultures were excluded. The primary outcome was a composite of clinical failure, including persistent blood cultures or fever for 5 days and in-hospital mortality. Secondary outcomes included adherence to a treatment bundle (appropriate empiric/definitive antibiotics, echocardiography (ECHO), duration of treatment, and repeat blood cultures).

**Results:**

A total of 250 patients were included. IDC was obtained in 62.0% of patients. More patients in the IDC group had endocarditis (20% vs 0%, p < 0.0001) and bone and joint infections (13.5% vs 1.1%, p = 0.001), compared to more UTI (16.8% vs 39.0%, p < 0.0001) in the non-IDC group. Patients in the IDC group had more murmurs on initial exam (21.3% vs 6.3%, p = 0.002), prosthetic device (49.7% vs 27.4%, p = 0.001), and NOVA scores of ≥ 4 (40.6% vs 18.9%, p < 0.0001). Most infections were due to *E. faecalis* (78.4%) and most were susceptible to vancomycin and ampicillin at 90.4% and 92.4%, respectively. The composite of clinical failure occurred in 22.6% of patients with IDC and 16.8% in the non-IDC group (p=0.274). There was higher adherence to the treatment bundle in the IDC group (Figure 1). More patients in the IDC group were treated with ampicillin (47.1% vs 22.1%, p < 0.0001), and numerically more patients received treatment with vancomycin in the non-IDC group (17.4% vs 24.2%, p = 0.068). In the multivariate analysis, vasopressors were the only independent predictor of the primary outcome (OR 9.3, 95% CI 3.5-24.8, p < 0.0001).

Figure 1. Adherence to treatment bundle. IDC = infectious diseases consultation, Echo = echocardiogram, * = p < 0.05

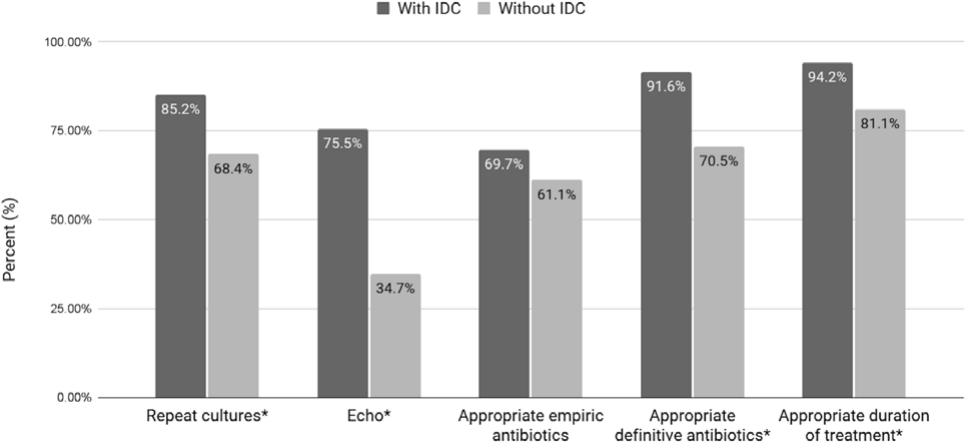

**Conclusion:**

There was no difference in rates of composite failure in patients with or without IDC; however, adherence to a treatment bundle was higher in the IDC group. IDC demonstrated stewardship benefits with regards to vancomycin usage.

**Disclosures:**

**All Authors**: No reported disclosures

